# International Multi-Site Initiative to Develop an MRI-Inclusive Nomogram for Side-Specific Prediction of Extraprostatic Extension of Prostate Cancer

**DOI:** 10.3390/cancers13112627

**Published:** 2021-05-27

**Authors:** Andreas G. Wibmer, Michael W. Kattan, Francesco Alessandrino, Alexander D. J. Baur, Lars Boesen, Felipe Boschini Franco, David Bonekamp, Riccardo Campa, Hannes Cash, Violeta Catalá, Sebastien Crouzet, Sounil Dinnoo, James Eastham, Fiona M. Fennessy, Kamyar Ghabili, Markus Hohenfellner, Angelique W. Levi, Xinge Ji, Vibeke Løgager, Daniel J. Margolis, Paul C. Moldovan, Valeria Panebianco, Tobias Penzkofer, Philippe Puech, Jan Philipp Radtke, Olivier Rouvière, Heinz-Peter Schlemmer, Preston C. Sprenkle, Clare M. Tempany, Joan C. Vilanova, Jeffrey Weinreb, Hedvig Hricak, Amita Shukla-Dave

**Affiliations:** 1 Department of Radiology, Memorial Sloan Kettering Cancer Center, 1275 York Avenue, New York, NY 10065, USA; hricakh@mskcc.org (H.H.); davea@mskcc.org (A.S.-D.); 2 Department of Quantitative Health Sciences in the Lerner Research Institute, Cleveland Clinic, Cleveland, OH 44195, USA; kattanm@ccf.org (M.W.K.); JIX@ccf.org (X.J.); 3 Department of Radiology, Brigham and Women’s Hospital, Harvard Medical School, Boston, MA 02115, USA; falessandrino@med.miami.edu (F.A.); Franco_Boschini@DFCI.HARVARD.EDU (F.B.F.); ffennessy@bwh.harvard.edu (F.M.F.); ctempany@bwh.harvard.edu (C.M.T.); 4 Charité University Hospital, 10117 Berlin, Germany; alexander.baur@charite.de (A.D.J.B.); Hannes.cash@charite.de (H.C.); tobias.penzkofer@charite.de (T.P.); 5 Herlev Gentofte University Hospital, 2730 Herlev, Denmark; Lars.Ploug.Boesen@regionh.dk (L.B.); Vibeke.Loegager@regionh.dk (V.L.); 6 DKFZ German Cancer Research Center, 69120 Heidelberg, Germany; d.bonekamp@dkfz-heidelberg.de (D.B.); janphilipp.radtke@uk-essen.de (J.P.R.); h.schlemmer@Dkfz-Heidelberg.de (H.-P.S.); 7 Department of Radiological Sciences, Oncology & Pathology, Sapienza University of Rome, 00185 Rome, Italy; riccardocampa23@gmail.com (R.C.); valeria.panebianco@uniroma1.it (V.P.); 8 Department of Urology, University Magdeburg, 39120 Magdeburg, Germany; 9 Department of Radiology, Fundació Puigvert, 08025 Barcelona, Spain; violetacatala@yahoo.com.ar; 10 Department of Uro-Radiology, Creu Blanca, 08034 Barcelona, Spain; 11 Hospices Civils de Lyon, Hôpital Edouard Herriot, 69003 Lyon, France; sebastien.crouzet@chu-lyon.fr (S.C.); paul-cezar.moldovan@chu-lyon.fr (P.C.M.); olivier.rouviere@netcourrier.com (O.R.); 12 Genitourinary and Women’s Imaging Departments, Lille University Hospital, 59037 Lille, France; sounildinnoo@gmail.com (S.D.); dicomworks@gmail.com (P.P.); 13 Department of Surgery, Memorial Sloan Kettering Cancer Center, New York, NY 10065, USA; easthamj@mskcc.org; 14 Department of Urology, Yale School of Medicine, New Haven, CT 06510, USA; kamyar.ghabili@yale.edu (K.G.); Preston.Sprenkle@yale.edu (P.C.S.); 15 Department of Urology, University Hospital of Heidelberg, 69120 Heidelberg, Germany; markus.hohenfellner@med.uni-heidelberg.de; 16 Department of Pathology, Yale School of Medicine, New Haven, CT 06510, USA; angelique.levi@yale.edu; 17 Weill Cornell Medicine, Weill Cornell Imaging, New York-Presbyterian Hospital, New York, NY 10021, USA; djm9016@med.cornell.edu; 18 Berlin Institute of Health (BIH), 10178 Berlin, Germany; 19 Faculté de Médecine Lyon Est, Université de Lyon, 69003 Lyon, France; 20 Clínica Girona, Institute Catalan of Health-IDI, University of Girona, 17004 Girona, Spain; kvilanova@comg.cat; 21 Department of Radiology, Yale School of Medicine, New Haven, CT 06510, USA; jeffrey.weinreb@yale.edu

**Keywords:** prostate cancer, clinical staging, extraprostatic tumor extension, magnetic resonance imaging, nomogram

## Abstract

**Simple Summary:**

For patients with newly diagnosed prostate cancer, it is important to detect tumor growth beyond the prostate, as this can affect a patient’s prognosis, influence management decisions, and alter treatment strategies. It is recognized that on prostate MRI, some instances of extraprostatic tumor growth can be missed. In this study, we merged patient data from multiple hospitals in different countries and developed a type of mathematical formula called “nomogram” that combines MRI findings with other available patient data. The results of our study allow physicians to more accurately diagnose extraprostatic tumor growth by combining clinical, biopsy, and MRI-derived information according to their relative statistical importance.

**Abstract:**

Background: To develop an international, multi-site nomogram for side-specific prediction of extraprostatic extension (EPE) of prostate cancer based on clinical, biopsy, and magnetic resonance imaging- (MRI) derived data. Methods: Ten institutions from the USA and Europe contributed clinical and side-specific biopsy and MRI variables of consecutive patients who underwent prostatectomy. A logistic regression model was used to develop a nomogram for predicting side-specific EPE on prostatectomy specimens. The performance of the statistical model was evaluated by bootstrap resampling and cross validation and compared with the performance of benchmark models that do not incorporate MRI findings. Results: Data from 840 patients were analyzed; pathologic EPE was found in 320/840 (31.8%). The nomogram model included patient age, prostate-specific antigen density, side-specific biopsy data (i.e., Gleason grade group, percent positive cores, tumor extent), and side-specific MRI features (i.e., presence of a PI-RADSv2 4 or 5 lesion, level of suspicion for EPE, length of capsular contact). The area under the receiver operating characteristic curve of the new, MRI-inclusive model (0.828, 95% confidence limits: 0.805, 0.852) was significantly higher than that of any of the benchmark models (*p* < 0.001 for all). Conclusions: In an international, multi-site study, we developed an MRI-inclusive nomogram for the side-specific prediction of EPE of prostate cancer that demonstrated significantly greater accuracy than clinical benchmark models.

## 1. Introduction

The diverse natural history of localized prostate cancer makes accurate risk stratification a challenging but indispensable requirement for selecting the most appropriate management strategy for any individual patient. Along with other clinical, blood- and biopsy-derived biomarkers, clinical cancer stage on digital rectal examination, which takes into account the presence or absence of extraprostatic disease extension, plays an integral part in risk stratification. While multiple prospective studies and meta-analyses have shown that magnetic resonance imaging (MRI) reliably detects clinically significant prostate cancer [1,2], it lacks sensitivity for diagnosing extraprostatic disease extension (EPE) [3]. For example, a recent meta-analysis pooling data from 9796 patients yielded a sensitivity of just 57% [4]. It must be noted, however, that for most patients in this analysis only a limited MRI protocol was acquired (i.e., T2-weighted sequences only) and that recent ‘multiparametric’ MRI protocols yielded higher pooled sensitivities for EPE in subgroup analyses [4]. A lack of awareness of this limited diagnostic precision may even result in adverse clinical outcomes, as was suggested by studies conducted earlier in the history of clinical prostate MRI, where the acquisition of pre-operative MRI was associated with a higher rate of positive surgical margins [5,6]. Although subsequent prospective studies [7,8] and a meta-analysis [9] have since contradicted those unfavorable findings, MRI alone cannot be used to reliably exclude or diagnose EPE. Nevertheless, MRI does offer high specificity for EPE (e.g., 91% in the above-mentioned meta-analysis [4]). Integrating MRI-derived information with clinical information might therefore result in more precise clinical staging, as demonstrated in multiple single-institution studies of American [10,11,12,13], European [14,15,16], and Asian [17] populations, as summarized in Table 1. The single-center methodology of all these prior studies, however, limits their generalizability. This is so not only because radiologists from different institutions might recognize and interpret MRI findings differently [18], but also because patient selection and management may differ between institutions. In fact, the encouraging results found in the single-institution studies cited above [10,11,12,13,14,15,16,17] failed to be reproduced [19,20] or were only partially reproduced [21] by other research groups (Table 1).

Inspired by discussions held at the Global Summit for Prostate Cancer (organized by the AdMeTech Foundation), we compiled an international, multi-site dataset of patients who had undergone pre-prostatectomy MRI and used this dataset to develop a new nomogram for the side-specific prediction of EPE based on clinical, biopsy-, and MRI-derived information. We then compared the performance of this nomogram to that of established, non-MRI-inclusive models for predicting EPE of prostate cancer.

## 2. Materials and Methods

This was a multi-site retrospective study of consecutive patients with biopsy-proven prostate cancer who underwent dedicated multi-parametric MRI before radical prostatectomy. Each participating institution was invited to provide anonymized data on up to 100 consecutive patients going backwards in time from 31 December 2017. The study design and submission of anonymized patient data were approved by the institutional review boards of all participating institutions. Demographic and clinical variables were retrospectively extracted from the medical records and included patient age, clinical stage on digital rectal examination, serum level of prostate-specific antigen (PSA), and PSA density. Biopsy data was collected separately for the left and right sides and included the number of cores taken, the number of positive cores, the highest Gleason grade group, as well as the maximum absolute and relative cancer extent in a single core. MRIs were acquired and interpreted at the respective institution according to the Prostate Imaging Reporting and Data system version 2.0 (PI-RADSv2.0), which has been described in detail previously [22]. The diameter and capsular contact length of the largest and/or most suspicious MRI-visible lesion was measured by the radiologist. The likelihood of EPE was scored by the interpreting radiologist on a 5-point Likert scale separately for the left and right sides of the gland according to previously published criteria [23]. To mitigate potential inter-site variabilities in the assignment of Likert scores for EPE, we reduced the original 5-tiered EPE Likert score to a 3-point scale for the statistical analyses as follows: EPE Likert scores of 1 and 2 were classified as “negative” for EPE on MRI, scores of 3 and 4 as “equivocal,” and scores of 5 as “positive”. Side-specific presence or absence of EPE on prostatectomy specimens as documented in the pathology reports served as the reference standard. After anonymization, all data were submitted to the leading institution; no central MRI or pathology review was performed.

### 2.1. Statistical Considerations

Multivariate imputation was performed by chained equations for the variables, and a logistic regression model was used to predict the side-specific presence of EPE on prostatectomy specimens. A series of regression models were run whereby each variable with missing data was modeled conditional upon the other variables in the data. The modeling process was repeated for a number of cycles, with the imputation being updated at each cycle. At the end, the final imputations were retained, resulting in one imputed dataset [24]. All predictors of interest were added in the starting full model before model selection. A reduced model was created using a stepdown model reduction technique that identifies the best parsimonious model using the concordance index as a stopping criterion. Variables for which more than 50% of data points were missing were excluded from the analysis. Restricted cubic splines were used for PSA. To evaluate the performance of the proposed model, bootstrap resampling with 1000 repetitions was adopted to assess 95% confidence interval (CI) of the area under the receiver operating characteristic (AUROC) curve before the calibration curves and decision analysis curves [25] were assessed. For cross validation, each time one center was used as validation data and the other centers as development data.

### 2.2. Benchmark Comparisons

The performance of the MRI-inclusive nomogram developed in the present study was benchmarked against established models for the prediction of EPE that are based on clinical and biopsy data, i.e., the Memorial Sloan Kettering Cancer Center (MSKCC) “Pre-Radical Prostatectomy” nomogram, which is derived from the data of 11,552 patients treated at MSKCC and considers: patient’s age, PSA levels, clinical tumor stage, biopsy Gleason grades/scores, and the proportion of positive biopsy cores [26]; the updated Partin tables, which are based on data from 5629 men who underwent surgery at the Johns Hopkins Hospital and integrate PSA levels, biopsy Gleason score, and clinical stage [27]; a prospectively developed and validated multi-institutional model based on data from 6823 patients collected by the Belgian Cancer Registry which is based on PSA levels, clinical cancer stage, biopsy Gleason score, and the proportion of positive biopsy cores [28]; and a side-specific nomogram (PSA, clinical stage, biopsy Gleason sum, percent positive cores, percent cancer in biopsy core) developed in Germany with data from 1118 prostatectomy patients [29]. The first three benchmark models were developed and intended for prediction of EPE for the whole prostate, and we applied them in our dataset on a whole-gland basis.

## 3. Results

### 3.1. Study Population

Data on 848 patients were submitted from 10 institutions (three in the United States of America; two each in France and Germany; one each in Denmark, Italy, and Spain). Eight cases were excluded due to incomplete data regarding EPE on prostatectomy specimens, leaving data from 840 individuals for the final analyses. The median time from prostate biopsy to prostatectomy was 86 (IQR: 63–118) days and the median time from MRI to prostatectomy was 76 (IQR: 40–113) days. The MRI was performed before biopsy in 393 patients (46.8%; median interval: 26 days, IQR: 10, 45) and after the biopsy in 340 (40.5%; median interval: 53 days, IQR: 33, 77). One-hundred-and-seven patients (12.7%) underwent MRI and biopsy on the same day. Systematic transrectal ultrasound-guided prostate biopsies were performed in 819/840 patients (97.5%) and the median number of systematic biopsy cores was 12 (IQR: 10–12) per patient and 6 (IQR: 5–6) per prostate side. In 189/840 (22.5%) individuals, targeted biopsies were taken from the right side of the prostate, in 219/840 (26.1%) from the left, and in 98/840 (11.7%) from both sides, the midline prostate, or an unspecified location. Because the aim of this study was side-specific prediction of EPE and the side-specific data completeness for targeted biopsies was less than 50%, these biopsies were not included in the statistical analyses. All MRI scans comprised T1-, T2-, and diffusion-weighted sequences; additional dynamic contrast-enhanced sequences were acquired in 687/840 cases (81.8%) and MRI spectroscopy was performed in 96/840 cases (11.4%). EPE was present in 320/840 prostatectomy specimens (38.1%), and the side-specific prevalence of EPE on histopathology was 365/1680 (21.7%). Detailed descriptive statistics on demographic, clinical, biopsy, and MRI data, as well as the proportion of missing data, are listed in Table 2.

### 3.2. Inter-Site Variabilities

We observed significantly different distributions of demographic, clinical, and biopsy parameters between institutions, including patient age, PSA levels, PSA density, and cancer stage on digital rectal examination, percentage of positive cores, maximum tumor extent in a single core, and biopsy Gleason grade groups (*p* < 0.001 for all, Table 2). On MRI, different institutions reported significantly different distributions of PI-RADSv2 scores, median lesion size, and lengths of capsular contact (*p* < 0.001 for all). The proportion of patients classified as “negative for EPE” on MRI ranged between 25.0% and 78.6%; “equivocal” findings for EPE were reported in 15.3% to 60.4%; and EPE was thought to be “definitely present” in 2.5–15.9% of individuals (*p* < 0.001). This data is detailed for every institution in Table 2. We did not observe significant inter-institutional differences in the prevalence of EPE on prostatectomy specimens (range: 29.3–47.7%) or the diagnostic accuracy for EPE on MRI (AUROC range: 0.65–0.83).

### 3.3. Nomogram and Benchmarks

**Figure 1:**
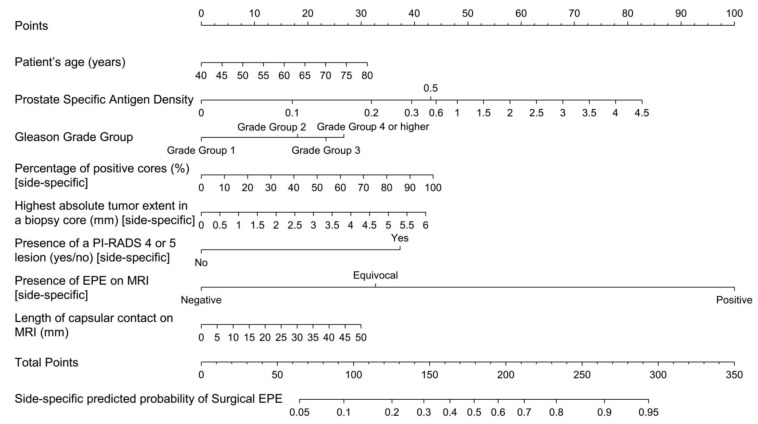
MRI-inclusive nomogram for the side-specific prediction of extraprostatic extension of prostate cancer.

**Figure 2:**
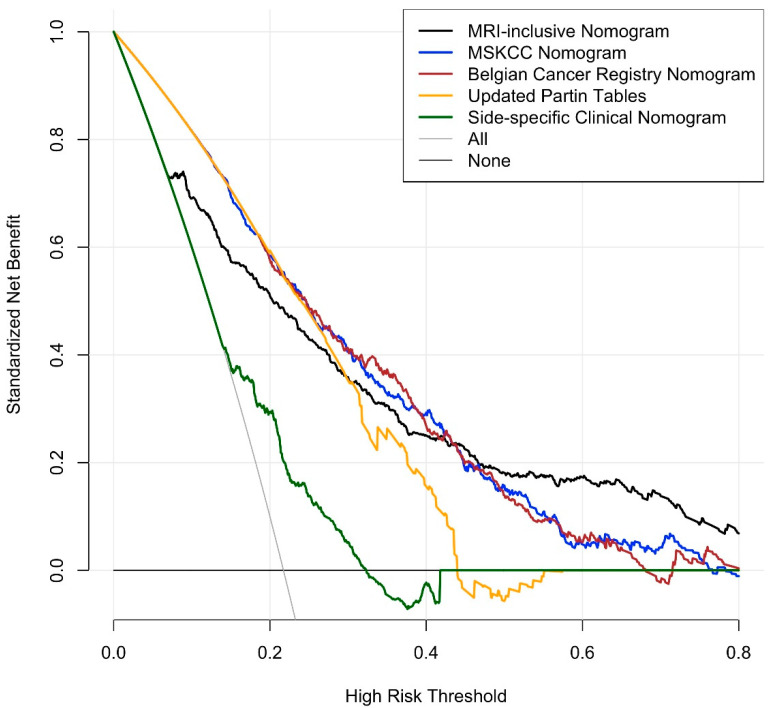
Decision curve analyses for the proposed MRI-inclusive nomogram and the benchmark models. The *y*-axis depicts the benefit of each nomogram to identify EPE correctly, and the *x*-axis refers to how clinicians appraise different outcomes in a given clinical context. A detailed guide for the interpretation of decision curves can be found in [30].

**Figure 3:**
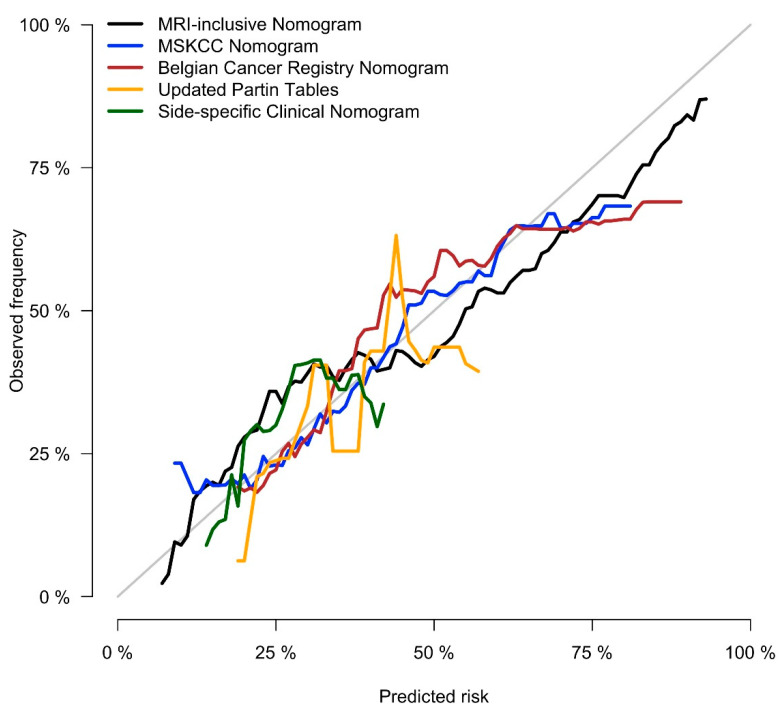
Calibration plot for the proposed MRI-inclusive nomogram and the benchmark models illustrating the actual frequency of EPE on the *y*-axis and the predicted probability on the *x*-axis (a more in-depth explanation can be found in [31]).

The initial model included patient age, PSA, PSA density, clinical tumor stage, side-specific biopsy data (i.e., percentage of positive systematic biopsy cores, highest Gleason grade group, largest tumor extent), and side-specific MRI data (i.e., presence of a PI-RADS 4/5 lesion, lesion diameter, level of suspicion for EPE, length of capsular contact). Clinical tumor stage, PSA, and lesion diameter on MRI were dropped through stepwise selection; the resulting nomogram included patient’s age, PSA density, as well as side-specific biopsy and MRI data, and detailed in Figure 1. Performance analysis yielded an AUROC of 0.828 for this model (bootstrap-validated 95% confidence limits: 0.805–0.852). Cross validation analyses, where each center was used as validation data and the other nine centers as development data, resulted in an average AUROC of 0.820 (range: 0.735–0.883). In our dataset, this new, MRI-inclusive model predicted EPE significantly more accurately than did any of the benchmark statistical models (*p* < 0.001 for all), as detailed in Table 3. Decision curve analyses of the proposed MRI-inclusive nomogram and benchmark models are displayed in Figure 2, and a calibration plot for all models in Figure 3.

To further validate the diagnostic performance of the proposed nomogram, we performed additional analyses by using data from six institutions as training set and data from the others as validation set. This process was repeated on all 210 possible permutations and yielded similar results as the bootstrap-validated model (AUROC: 0.821 vs. 0.828) (Table S1). The analyses were repeated without imputation of missing data and the AUROC of the MRI-inclusive nomogram was slightly lower compared with the bootstrap-validated model with imputed data (AUROC 0.799 vs. 0.828) (Table 4).

## 4. Discussion

In this international, multi-site study, we developed an MRI-inclusive nomogram for the side-specific prediction of EPE of prostate cancer. The nomogram integrates demographic, clinical, biopsy-, and MRI-derived variables and offers two advantages over established prediction models: First, it provides side-specific information about EPE, which is particularly useful for surgical or radiation therapy planning. Secondly, it predicts EPE more accurately than established statistical models and may help clinicians better assess a given patient’s risk for disease progression.

Our results corroborate findings from prior single-institution studies where the addition of MRI-derived information to clinical and biopsy data led to more precise predictions of EPE, both on a side-specific basis [11,14,15,17], as well as for the whole prostate [10,13,16]. However, clinical side-specific nomograms without MRI data were also found to be highly accurate in external validation cohorts [32], and other single-center studies did not reproduce the promising results of MRI-inclusive models. For example, in a retrospective study of 236 patients, the integration of MRI findings did not significantly increase the precision of the MSKCC pre-radical prostatectomy nomogram [19]. One of the reasons for the inconsistencies of published data might lie in the well-documented variability of radiologists’ performance levels in identifying EPE on MRI [33], a skill that strongly depends on dedicated training [34] and the degree of sub-specialization [35]. In fact, a prospective study including three radiologists showed that while two of them added incremental precision to clinical prostate cancer staging with their MRI interpretations, the third failed to do so [21]. Inter-site differences in patient selection and management strategies may also limit the reproducibility of single-center studies. In the above-cited studies [10,11,12,13,14,15,16,17,19,20,21], for example, the proportion of patients with extraprostatic disease extension on digital rectal examination (i.e., cT3) and on prostatectomy specimens (i.e., pT3a) ranged between 0–21% and 16–55%, respectively, and the percentage of patients with a Gleason score of 8 or higher on biopsy ranged from 2.7% to 44.0%. These data closely resemble data from our study cohort, where the frequency of cT3 disease ranged between 0% and 10%, and the proportion of patients with a biopsy Gleason score ≥8 ranged from 9.0% to 44.8% among institutions. These figures highlight the substantial inter-site differences in patient selection for prostatectomy. Consequently, single-institution cohorts may lack representativeness, making statistical models derived from them challenging to reproduce. This challenge points to the importance of pooling international multi-site data—as was done in the present study—for creating more comprehensive and representative datasets and thus increasing the robustness and external applicability of any deduced statistical model.

The performance comparisons between the nomogram developed in this study and the established benchmark models must be interpreted carefully. The proposed MRI-inclusive nomogram might be overfitted and its performance might be overestimated despite bootstrap- and cross-validation. The benchmark models performed similarly or slightly worse in our study cohort than in their respective training cohorts. While the MSKCC pre-radical prostatectomy nomogram performed equally well in our study cohort as it did in its development cohort (i.e., AUROC 0.675 vs. 0.657), the updated Partin tables model was slightly less accurate in our cohort than in its development cohort (i.e., AUROC 0.601 vs. 0.702); this might have been due to differences in the two patient cohorts, with the Johns Hopkins prostatectomy cohort having, on average, lower-risk disease, as exemplified by the lower prevalence of biopsy Gleason score ≥8 (i.e., 8% vs. 19%) and EPE on prostatectomy specimens (i.e., 23% vs. 38%) [27]. Similarly, the Belgian Cancer Registry model performed slightly less well in the present cohort than in its development cohort (AUROC 0.679 vs. 0.773); this model was also developed in a population with lower-risk disease than the present cohort (e.g., biopsy Gleason score ≥8: 12% vs. 19%; EPE on prostatectomy: 19% vs. 38%) [28]. The side-specific clinical benchmark model by Steuber et al. performed less accurately in the current than the original cohort (AUROC: 0.650 vs. 0.840) [29]. Here again, the risk profile was different between the cohorts, as exemplified by the proportion of patients with T1c disease (82% vs. 69%) or pathological EPE (27% vs. 38%) [29]. Even taking all these potential confounders into account, we infer that the separation of the ROC curves is wide enough to conclude that the MRI-inclusive nomogram presented herein predicts EPE more accurately than do established clinical prediction models.

The current study corroborates the high specificity and positive predictive value of prostate MRI for the diagnosis of EPE [3], as reflected by the relative weight of a ‘positive MRI’ in our nomogram. It also highlights the similar relative importance of clinical and biopsy-derived metrics, as exemplified by the high statistical weight of PSA density and biopsy tumor amount in the nomogram. In high-risk patients with unequivocal EPE on MRI, the nomogram might therefore provide only limited additional information. However, in less definitive cases, for example those with equivocal MRI findings and/or low/intermediate risk features, the statistically appropriate integration of multiple datapoints through this nomogram might help to more accurately assess the likelihood of EPE. The utility of MRI as a component of local staging tools might be further increased by the extraction of radiomic data in combination with machine learning or artificial intelligence algorithms, as suggested by recent studies [36,37,38,39]. The main limitation of our study is its retrospective design and the fact that all patients underwent prostatectomy introduces a selection bias. Although it is possible that the identification and interpretation of MRI findings differed between institutions [18], our pooled data likely provides a balanced representation of current radiology practice patterns from multiple countries. The incompleteness of side-specific data on targeted biopsies is another limitation of this study as MRI-targeted biopsies increase the detection rate of high-grade cancer [40]; given the association of high-grade cancer and EPE in our and multiple previously published cohorts, this higher detection rate would likely translate into a more accurate prediction of EPE. As discussed above, our statistical model might be overfitted and might perform worse in external validation cohorts. MRIs and prostatectomy specimens were reviewed by a single radiologist/pathologist at each institution and we did not assess for inter-reader variability, which is documented in the literature for radiologists [41,42] and pathologists [43,44].

## 5. Conclusions

This study produced a new MRI-inclusive nomogram for side-specific prediction of EPE of prostate cancer based on data from ten sites in six countries. The nomogram integrates demographic, clinical, biopsy, and MRI data and outperforms clinical benchmark models.

## Figures and Tables

**Table 1:** Summary of previously published single-center studies investigating whether the addition of MRI information to established clinical/biopsy predictors of EPE could increase predictive accuracy.

Authors	Country	No.	Whole Gland vs. Side-Specific	MRI Variables	Benchmark Models/ Benchmark Clinical Data	Main Finding
Rayn et al. [10]	USA	532	Whole gland	NIH suspicion score EPE: present vs. absent Largest lesion diameter	MSKCC nomogram Partin tables	MRI in addition to clinical nomograms increases predictive ability.
Martini et al. [11]	USA	561	Side-specific	EPE: absent vs. present	PSA, Bx Gleason grade group, % cancer in Bx cores	MRI-inclusive model for the side-specific prediction of EPE.
Morlacco et al. [12]	USA	501	Whole gland	EPE: absent vs. present	Partin tables CAPRA score	MRI-inclusive models outperform clinical-based models alone.
Feng et al. [13]	USA	112	Whole gland	EPE: absent vs. present	MSKCC nomogram Partin tables	MRI improved accuracy of existing clinical nomograms.
Zapala et al. [14]	Poland	88	Side-specific	Likert score (1–5) EPE: present vs. absent Largest lesion diameter	PSA, cT, number and % positive Bx cores, % cancer in Bx cores, Bx Gleason score	Lesion diameter ≥ 15 mm on MRI is an independent predictor of EPE.
Nyarangi-Dix et al. [15]	Germany	264	Side-specific	EPE: ESUR score (1–5) Prostate volume Capsular contact length	MSKCC nomogram Nomogram by Steuber et al.	Combining MRI and clinical parameters outperformed clinical nomograms.
Lebacle et al. [16]	France	1743	Whole gland	EPE: present vs. absent	PSA, Gleason score, prostate weight, cT	MRI-inclusive model is more accurate than clinical and biopsy data alone.
Chen et al. [17]	China	706	Side-specific	EPE risk score (1–5)	Age, cT, PSA, Bx Gleason grade groups, % positive Bx cores, % cancer in bx cores	MRI-inclusive model is more accurate than clinical and biopsy data alone.
Weaver et al. [19]	USA	236	Whole gland	PI-RADS score EPE: present vs. absent	MSKCC nomogram	A combined model (MRI + MSKCC) provides no additional benefit over the MSKCC nomogram alone.
Jansen et al. [20]	Netherlands	430	Whole gland	EPE: present vs. absent	MSKCC nomogram Partin tables	The addition of MRI to the MSKCC and Partin nomograms did not increase diagnostic accuracy.
Zanelli et al. [21]	Italy	73	Whole gland	PI-RADS score EPE: ESUR score (1–5)	MSKCC nomogram CAPRA score	Combination of MRI + clinical models outperforms clinical models for two radiologists, but not for a third.

Bx, biopsy; CAPRA, Cancer of the Prostate Risk Assessment; cT, clinical T-stage; EPE, extraprostatic extension; ESUR, European Society of Urogenital Radiology; MRI, magnetic resonance imaging; MSKCC, Memorial Sloan Kettering Cancer Center; NIH, National Institute of Health; No, number of patients; PI-RADS, Prostate Imaging Reporting and Data System; PSA, prostate-specific antigen; USA, Unites States of America.

**Table 2:** Clinical, biopsy, and MRI-derived data for the entire study cohort and every participating institution. Continuous variables are presented as medians (interquartile range); # for inter-institutional comparisons; * only data on relative core involvement submitted, # *p*-values for inter-institutional comparisons.

Parameter		Overall	Institution										
			A	B	C	D	E	F	G	H	I	J	*p*-Value ^#^
Number of Patients		840	100	82	98	96	100	44	100	100	20	100	
**Clinical Data**	Age (years)	64.0 [59.0, 68.0]	60.5 [55.0, 66.0]	66.0 [61.0, 69.0]	59.0 [53.3, 64.0]	66.0 [62.0, 71.0]	65.0 [62.0, 67.3]	65.5 [58.8, 73.3]	65.0 [60.0, 68.0]	63.5 [60.0, 66.0]	65.0 [63.0, 68.0]	63.0 [59.0, 68.0]	<0.001
	PSA (ng/mL) Missing: 0.1%	7.1 [5.2, 10.7]	6.1 [4.5, 8.3]	7.9 [5.7, 10.1]	5.4 [4.3, 7.6]	9.0 [6.5, 14.2]	11.8 [7.3, 18.0]	8.3 [5.2, 13.9]	6.0 [5.0, 9.0]	6.4 [5.2, 8.8]	7.3 [6.1, 9.6]	7.2 [5.4, 10.4]	<0.001
	PSA Density (ng/mL^2^) Missing: 0.2%	0.2 [0.1, 0.3]	0.2 [0.1, 0.3]	0.1 [0.1, 0.3]	0.2 [0.1, 0.3]	0.2 [0.1, 0.3]	0.3 [0.2, 0.4]	0.2 [0.1, 0.3]	0.1 [0.1, 0.2]	0.2 [0.1, 0.2]	0.2 [0.1, 0.3]	0.2 [0.1, 0.3]	<0.001
**Systematic Biopsy Data**	Positive Biopsy Cores (%) Missing: 3.2%	25.0 [12.5, 41.7]	35.9 [21.4, 59.8]	16.7 [8.3, 41.7]	41.7 [25.0, 54.6]	30.0 [18.3, 50.0]	20.0 [10.0, 40.0]	16.7 [9.9, 35.7]	25.0 [8.3, 41.7]	25.0 [10.0, 40.0]	33.3 [31.7, 46.7]	16.7 [8.3, 33.3]	<0.001
	Highest Gleason Grade Group												
	1	216 (25.7)	18 (18.0)	17 (20.7)	26 (26.5)	22 (22.9)	36 (36.0)	8 (18.2)	26 (26.0)	33 (33.0)	0	30 (30.0)	<0.001
	2	293 (34.9)	45 (45.0)	35 (42.7)	44 (44.9)	20 (20.8)	29 (29.0)	11 (25.0)	40 (40.0)	33 (33.0)	0	36 (36.0)	
	3	97 (11.5)	12 (12.0)	4 (4.9)	19 (19.4)	6 (6.2)	6 (6.0)	8 (18.2)	11 (11.0)	14 (14.0)	0	17 (17.0)	
	4 or higher	163 (19.4)	23 (23.0)	21 (25.6)	9 (9.2)	43 (44.8)	16 (16.0)	13 (29.5)	14 (14.0)	9 (9.0)	3 (15.0)	12 (12.0)	
	Cancer only on targeted biopsy	71 (8.5)	2 (2.0)	5 (6.1)	0	5 (5.2)	13 (13.0)	4 (9.1)	9 (9.0)	11 (11.0)	17 (85.0)	5 (5.0)	<0.001
	Maximum tumor extent (mm) Missing: 11.9%	4.0 [1.5, 8.0]	6.0 [3.0, 9.0]	4.0 [1.0, 12.0]	Missing *	5.0 [2.0, 8.0]	3.0 [2.0, 8.0]	1.0 [0.0, 5.0]	5.0 [3.0, 9.0]	4.0 [2.0, 7.0]	1.6 [1.6, 1.80]	5.0 [2.0, 7.0]	<0.001
**Data**	Highest PI-RADS score												
	1	9 (1.1)	0	0	0	0	0	0	5 (5.0)	0	0	4 (4.0)	<0.001
	2	31 (3.7)	10 (10.0)	7 (8.5)	4 (4.1)	0	1 (1.0)	0	2 (2.0)	6 (6.0)	0	1 (1.0)	
	3	83 (9.9)	11 (11.0)	14 (17.1)	9 (9.2)	4 (4.2)	9 (9.0)	6 (13.6)	11 (11.0)	6 (6.0)	2 (10.0)	11 (11.0)	
	4	339 (40.4)	31 (31.0)	26 (31.7)	55 (56.1)	41 (42.7)	34 (34.0)	17 (38.6)	38 (38.0)	45 (45.0)	13 (65.0)	39 (39.0)	
	5	378 (45.0)	48 (48.0)	35 (42.7)	30 (30.6)	51 (51.1)	56 (56.0)	21 (47.7)	44 (44.0)	43 (43.0)	5 (25.0)	45 (45.0)	
	Cases with PI-RADSv2 ≥ 4	717 (85.4)	79 (79.0)	61 (74.4)	85 (86.7)	92 (95.8)	90 (90.0)	38 (86.4)	82 (82.0)	88 (88.0)	18 (90.0)	84 (84.0)	0.005
	Maximum Lesion Diameter (cm)	1.5 [1.1, 2.0]	1.6 [1.2, 2.2]	1.4 [1.0, 2.1]	1.3 [1.0, 1.6]	1.6 [1.1, 2.4]	1.6 [1.2, 2.3]	1.5 [1.2, 2.2]	1.3 [1.0, 1.9]	1.6 [1.2, 2.0]	1.2 [1.0, 1.5]	1.4 [1.0, 1.8]	<0.001
	Length of Capsular Contact (mm)	10.0 [4.0, 17.0]	12.0 [4.0, 23.3]	13.0 [8.0, 20.0]	8.0 [5.0, 12.0]	16.0 [8.8, 25.0]	7.0 [2.0, 13.5]	13.0 [7.8, 22.3]	8.0 [0.0, 14.0]	15.0 [11.0, 20.0]	10.5 [7.8, 14.3]	0.0 [0.0, 12.0]	<0.001
	Presence of ECE												
	Negative	487 (58.0)	47 (47.0)	50 (61.0)	77 (78.6)	33 (34.4)	55 (55.0)	11 (25.0)	71 (71.0)	63 (63.0)	5 (25.0)	75 (75.0)	<0.001
	Equivocal	284 (33.8)	42 (42.0)	30 (36.6)	15 (15.3)	58 (60.4)	36 (36.0)	26 (59.1)	18 (18.0)	26 (26.0)	12 (60.0)	21 (21.0)	
	Positive	69 (8.2)	11 (11.0)	2 (2.5)	6 (6.1)	5 (5.2)	9 (9.0)	7 (15.9)	11 (11.0)	11 (11.0)	3 (15.0)	4 (4.0)	
ECE on prostatectomy specimen (standard of reference)		320 (38.1)	42 (42.0)	24 (29.3)	28 (28.6)	34 (35.4)	35 (35.0)	21 (47.7)	44 (44.0)	43 (43.0)	6 (30.0)	43 (43.0)	0.136

**Table 3:** Performance statistics of the MRI-inclusive nomogram and the benchmark models.

Statistical Model	Area under the Receiver Operator Characteristics Curve (95% Confidence Intervals)
MRI-inclusive Nomogram	0.828 (0.805, 0.852)
MSKCC Pre-Radical Prostatectomy Nomogram [26]	0.675 (0.638, 0.712) *
Belgian Cancer Registry Nomogram [28]	0.679 (0.641, 0.716) *
Updated Partin Tables [27]	0.601 (0.563, 0.640) *
Side-Specific Clinical Nomogram [29]	0.650 (0.619, 681) *

* *p*-value < 0.001 for comparison with the MRI-inclusive nomogram.

**Table 4:** Validation of the diagnostic performance of the MRI-inclusive and benchmark nomograms by using data from six institutions as training set and data from the others as validation set. This process was repeated on all 210 possible permutations, both with and without imputation of missing data.

Nomogram Model	Mean Area under the Receiver Operator Characteristics Curve (Range)	
	With Imputation *	Without Imputation *
MRI-inclusive Nomogram	0.821 (0.762, 0.880)	0.799 (0.738, 0.857)
MSKCC Pre-Radical Prostatectomy Nomogram [26]	0.678 (0.605, 0.725)	0.684 (0.587, 0.806)
Belgian Cancer Registry Nomogram [28]	0.681 (0.599, 0.731)	0.684 (0.599, 0.777)
Updated Partin Tables [27]	0.600 (0.533, 0.678)	0.607 (0.536, 0.708)
Side-Specific Clinical Nomogram [29]	0.652 (0.585, 0.727)	0.626 (0.535, 0.727)

* with/without imputation of missing data.

## Data Availability

The Electronic Health Record data in this study are not publicly available.

## Supplementary Materials

The following are available online at https://www.mdpi.com/article/10.3390/cancers13112627/s1, Table S1: Validation of the diagnostic performance of the MRI-inclusive and benchmark nomograms using data from six institutions as training set and data from the others as validation set. This process was repeated on all 210 possible permutations, both with and without imputation of missing data.
