# Supplementary material for: International Multi-Site Initiative to Develop an MRI-Inclusive Nomogram for Side-Specific Prediction of Extraprostatic Extension of Prostate Cancer
Source: Cancers (Basel). 2021 May 27;13(11):2627. doi: 10.3390/cancers13112627 (PMC8198352; doi:10.3390/cancers13112627)
Supplement: Supplementary file 1 [file cancers-13-02627-s001.zip › cancers-1187481-supplementary.pdf]

cancers

MDPI

Supplementary Materials: International Multi-Site Initiative to Develop an MRI-Inclusive Nomogram for Side-Specific Prediction of Extraprostatic Extension of Prostate Cancer

Andreas G. Wibmer, Michael W. Kattan, Francesco Alessandrino, Alexander D. J. Baur, Lars Boesen, Felipe Boschini Franco, David Bonekamp, Riccardo Campa, Hannes Cash, Violeta Catalá, Sebastien Crouzet, Sounil Dinnoo, James Eastham, Fiona M. Fennessy, Kamyar Ghabili, Markus Hohenfellner, Angelique W. Levi, Xinge Ji, Vibeke Løgager, Daniel J. Margolis, Paul C. Moldovan, Valeria Panebianco, Tobias Penzkofer, Philippe Puech, Jan Philipp Radtke, Olivier Rouvière, Heinz-Peter Schlemmer, Preston C. Sprenkle, Clare M. Tempany, Joan C. Vilanova, Jeffrey Weinreb, Hedvig Hricak and Amita Shukla-Dave

Table S1. Validation of the diagnostic performance of the MRI-inclusive and benchmark nomograms by using data from six institutions as training set and data from the others as validation set. This process was repeated on all 210 possible permutations, both with and without imputation of missing data.

**Nomogram Model**	**Mean Area under the Receiver Operator Characteristics Curve (range)**	
	With imputation*	Without imputation*
MRI-inclusive Nomogram	0.821 (0.762, 0.880)	0.799 (0.738, 0.857)
MSKCC Pre-Radical Prostatectomy Nomogram [26]	0.678 (0.605, 0.725)	0.684 (0.587, 0.806)
Belgian Cancer Registry Nomogram [28]	0.681 (0.599, 0.731)	0.684 (0.599, 0.777)
Updated Partin Tables [27]	0.600 (0.533, 0.678)	0.607 (0.536, 0.708)
Side-specific Clinical Nomogram [29]	0.652 (0.585, 0.727)	0.626 (0.535, 0.727)

* with/without imputation of missing data
